# Kindler syndrome: a rare case report from Syria

**DOI:** 10.1097/MS9.0000000000000503

**Published:** 2023-04-06

**Authors:** Souma Edrees, Natalie Jarkas, Munawar Hraib, Khaled Al-Yousef, Roula Baddour

**Affiliations:** Departments of a Dermatology; b Urology, Tishreen University Hospital; c Faculty of Medicine, Tishreen University, Latakia, Syria

**Keywords:** Case report, epidermolysis bullosa, hypertrichosis, Kindler syndrome, photosensitivity, poikiloderma

## Abstract

Kindler syndrome is a rare autosomal recessive inherited disease. The authors report a case with unique presentation that has never reported before in the medical Literatur” lanugo hair”. This is a case of a 13-year-old Syrian child, who presented with difuse fine face hair, and serious urinary complications. Kindler syndrome is characterized by acral skin blistering beginning at birth, diffuse cutaneous atrophy, photosensitivity, poikiloderma, and various mucosal findings. Highlighting a set of clinical diagnostic criteria; which is used only if a genetic test is not available

## Introduction

Highlights

- Kindler syndrome (KS) is a rare autosomal recessive genodermatosis.
- KS is an inherited syndrome manifested by acral blistering and photosensitivity in infancy and early childhood (decreasing after adolescence), followed by skin atrophy (cigarette-paper skin), and poikiloderma (persisting). Mucosal lesions are common and may lead to severe disability, because of stenosis of the mucosal cavities.
- KS can be diagnosed by clinical findings and detecting FERMT1 gene mutation.
- In 2005, Angelova–Fischer and colleagues proposed set of clinical diagnostic criteria; five major (acral blistering in infancy and childhood, Progressive poikiloderma, skin atrophy, abnormal photosensitivity, and gingival fragility, and/ or swelling), two minor (syndactyly, mucosal involvement: urethral, anal, oesophageal, and laryngeal stenosis), as well as associated findings for the diagnosis (nail dystrophy, ectropion of the lower lid, palmoplantar keratoderma, pseudoainhum, leucokeratosis of the lips, squamous cell carcinoma, anhidrosis/hypohidrosis-skeletal abnormalities, poor dentition/dental caries/periodontitis).

KS is a rare autosomal recessive genodermatosis, first described in 1954 by Theresa Kindler^1^. It is a rare subtype of hereditary epidermolysis bullosa, caused by a mutation in FERMT1, the gene that encodes a kindlin-1 protein, and is expressed in the skin, periodontal tissue, and bowel^2^. Only 250 cases have been reported globally. KS is characterized by a combination of features of acral skin blistering beginning at birth, diffuse cutaneous atrophy, photosensitivity, and poikiloderma. Mucosal findings are also common, including haemorrhagic mucositis, periodontal disease, premature loss of teeth, labial leukokeratosis, ectropion, and urethral stenosis^2^,^3^. We present a case of KS in a Syrian child.

## Case presentation

**Figure 1:**
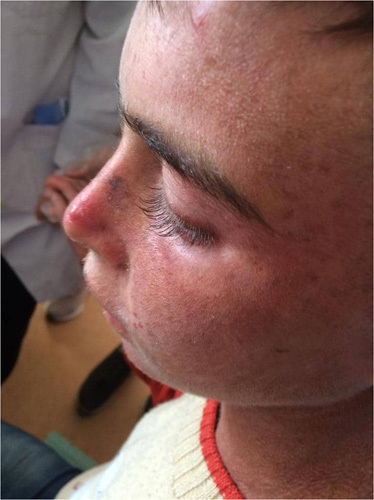
Clinical manifestations: hypertrichosis of lanugo hair in the face with photosensitivity.

A 13-year-old child, born to consanguineous, skin-healthy parents, presented to our Department with a history of skin fragility, recurrent blisters in the sites of trauma, photosensitivity, and hypertrichosis of lanugo hair mainly in the face since birth (Fig. 1). He also suffered from mild dysphagia and urethral stenosis with a resulting dysuria. His older sister had similar findings. He was diagnosed with epidermolysis bullosa soon after birth, as he started to have many blisters at the age of 4 months. Blisters formation decreased with age.

His medical history revealed anaemia, and many urological surgeries since the age of 5 years. He had many urethral dilation operations because of urethral stenosis. The urology department diagnosed him with urethral stenosis, vesicoureteral reflux, and cystic diverticulum. Ureteral replanting and urethral dilation were performed with good outcomes.

**Figure 2:**
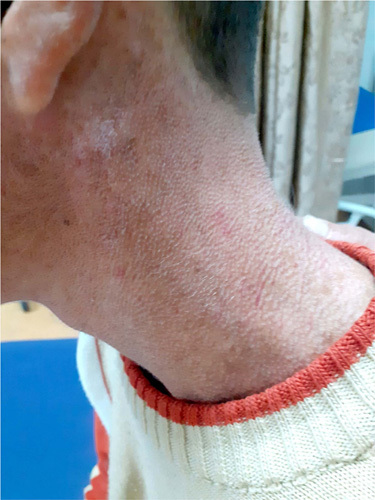
Poikiloderma: manifested by hypopigmented and hyperpigmented macules, reticular telangiectasia, and epidermal atrophy of sun exposed skin.

**Figure 3:**
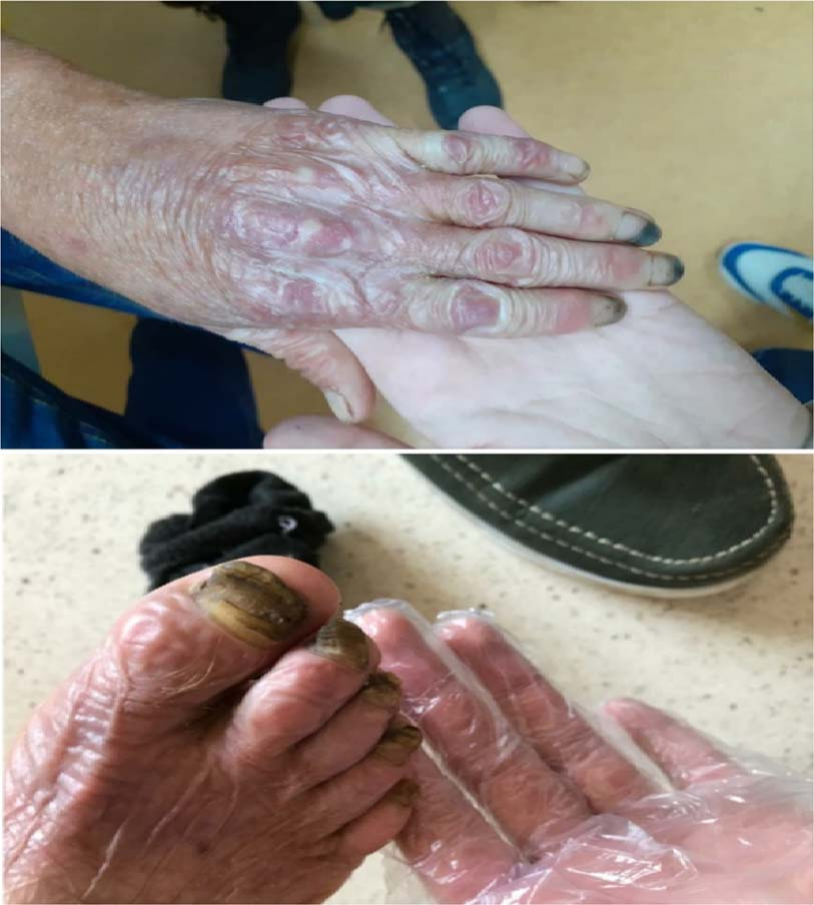
Skin atrophy-cigarette-paper-like wrinkled appearance on the dorsal surface of the hand with long and thickened cuticles, pseudosyndyctyly, and nail dystrophy.

**Figure 4:**
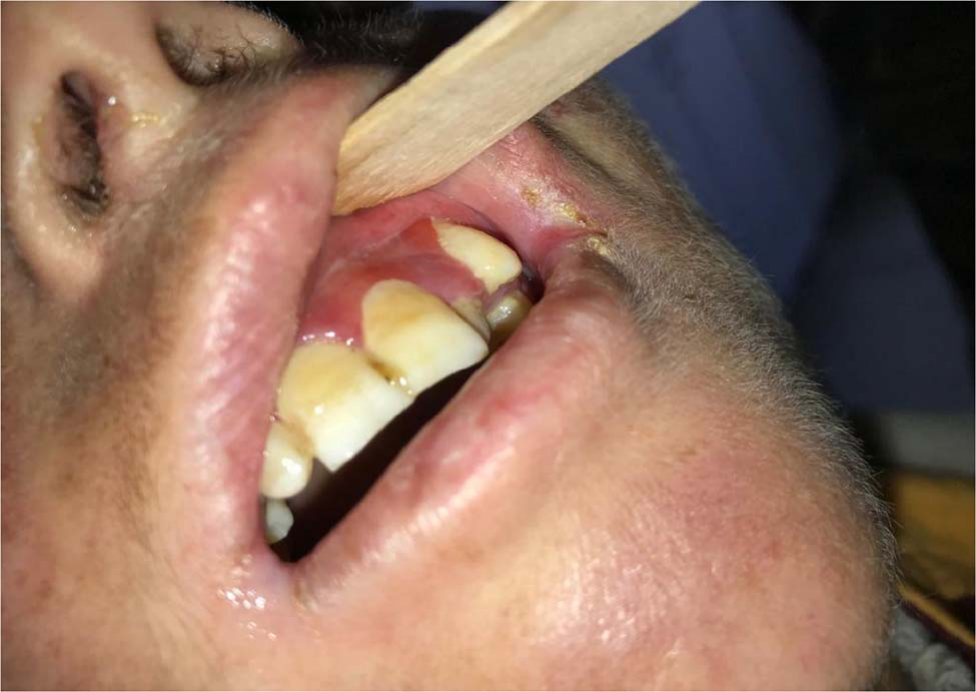
Gingival erosions and swelling, limited oral opening with angular cheilitis.

Physical examination revealed xerosis, poikiloderma of the face upper chest hands and feet (Fig. 2), with a cigarette-paper-like appearance of the hand and feet (Fig. 3), pseudosyndactyly, nail dystrophy of feet, adermatoglyphia of hands, and long cuticles. Examination of the oral cavity showed erosive stomatitis, recurrent bleeding, and limited oral opening with angular cheilitis (Fig. 4).

### Ophthalmologic investigation revealed xerophthalmia and conjunctivitis

Histopathological examination of a biopsy taken from an atrophic skin lesion revealed nonspecific features with atrophic epidermis, hydropic changes in the basal cell layer, melanin incontinence, and perivascular lymphocytic infiltrate.

Based on clinical and histopathological findings, the diagnosis of KS was confirmed.

Symptomatic treatment was given to the patient with advice to avoid trauma and direct exposure to sunlight.

## Discussion

KS is an inherited syndrome manifested by acral blistering and photosensitivity in infancy and early childhood (decreasing after adolescence), followed by skin atrophy (cigarette-paper skin), and poikiloderma (persisting). Mucosal lesions are common and may lead to severe disability, because of stenosis of the mucosal cavities. Other features include pseudosyndactyly, ainhum, nail dystrophy, palmoplantar keratoderma, ectropion, keratoconjunctivitis, conjunctival scarring, and anaemia^2^–^4^. Malignant skin tumours like squamous cell carcinoma can be developed in 10% of patients after age of 45 years^1^,^2^.

KS has been described mainly in Arab individuals, as well as Indians, Iranians, Turks, and Europeans^4^.

The histopathological features in light microscopy are nonspecific, include attenuated and flattened epidermis, hydropic alteration with cleft formation, and colloid bodies. The dermis contains dilated vessels, with dermal oedema and moderate fibrosis^5^. Transmission electron microscopy reveals major disorganization of the basement membrane, single or multiple cleavage planes at the level of the cutaneous basement membrane and reduplication of the lamina densa^4^.

KS can be diagnosed by clinical findings and detecting FERMT1 gene mutation^3^. The FERMT1 gene encodes the kindlin-1 protein, which is a part of focal adhesions that help bind actin filaments in basal keratinocytes to the underlying extracellular matrix^2^,^6^.

In 2005, Angelova–Fischer and colleagues proposed set of clinical diagnostic criteria;

Five major (acral blistering in infancy and childhood, progressive poikiloderma, skin atrophy, abnormal photosensitivity, and gingival fragility and/ or swelling), two minor (syndactyly, mucosal involvement: urethral, anal, oesophageal, laryngeal stenosis), as well as associated findings for the diagnosis (nail dystrophy, ectropion of the lower lid, palmoplantar keratoderma, pseudoainhum, leucokeratosis of the lips, squamous cell carcinoma, anhidrosis/hypohidrosis-skeletal abnormalities, poor dentition/dental caries/periodontitis). The diagnosis of KS is “certain” when four major criteria are present. The diagnosis is “probable” when three major and two minor criteria are present. If two major criteria and two minor criteria or associated symptoms are present, the diagnosis is considered to be “likely”^7^. The main criterion for diagnosis remains the analysis of the mutation in FERMT1 gene, and the above proposal is used only if a genetic test is not available, as in our case.

The differential diagnosis of KS may include; Bloom Syndrome (Congenital Telangiectatic Erythema), Cockayne Syndrome, Dyskeratosis Congenita, Epidermolysis Bullosa, Rothmund-Thomson Syndrome, and Xeroderma Pigmentosum^4^.

Here, we report a case in a Syrian child, who presented four major and one minor of the proposed clinical diagnostic criteria and two associated findings.

To our knowledge, this is the first time that “hypertrichosis of lanugo hair mainly in the face since birth” is mentioned in the clinical picture of KS in the medical literature, we suggest further studies, as it may be a symptom associated with KS.

Treatment of KS is mainly symptomatic includes standard blister care, avoiding trauma and sun exposure, and using moisturizers, with management of mucosal involvement and associated complications, as well as skin cancer screening^3^. Finally, we have to mention that this work has been reported in line with the SCARE 2020 criteria^8^.

## Conclusion

We have presented a KS case that displays a feature that was not described before (lanugo hair) and serious urinary complications. Dermatologists should be aware of the potential consequences of the syndrome especially regarding the genito-urinary system.

## Ethical approval

Not applicable, because this article does not contain any studies with human or animal subjects.

## Consent

Written informed consent was obtained from the patient family for publication of this case report and accompanying images. A copy of the written consent is available for review by the Editor-in-Chief of this journal on request.

## Sources of funding

This research received no specific grant from any funding agency in the public, commercial, or not-for-profit sectors.

## Author contribution

All authors contributed in all the phases of preparing the paper.

## Conflicts of interest disclosure

The authors declare that there is no conflict of interest.

## Registration of research studies

It’s a case report; not a clinical trial.

## Guarantor

Prof. Dr. Roula Baddour.

## Authorship

All authors attest that they meet the current ICMJE criteria for Authorship.

## Provenance and peer review

Not commissioned, externally peer-reviewed.
